# The mediating effect of self-efficacy on the relationship between self-care ability and disability level in older adult patients with chronic diseases

**DOI:** 10.3389/fpubh.2024.1442102

**Published:** 2024-09-13

**Authors:** Tiemei Wang, Senlin Wang, Nianwei Wu, Yan Liu

**Affiliations:** ^1^Department of General Surgery, Center for Obesity and Metabolic Health, The Third People's Hospital of Chengdu, The Affiliated Hospital of Southwest Jiaotong University, Chengdu, China; ^2^College of Medicine, Southwest Jiaotong University, Chengdu, China; ^3^Medical Research Center, The Third People's Hospital of Chengdu, The Affiliated Hospital of Southwest Jiaotong University, Chengdu, China; ^4^Research Center for Obesity and Metabolic Health, College of Medicine, Southwest Jiaotong University, Chengdu, China; ^5^Nursing Department, West China School of Public Health and West China Fourth Hospital, West China Nursing School, Sichuan University, Chengdu, Sichuan, China

**Keywords:** older adult, chronic diseases, self-efficacy, self-care, disability

## Abstract

**Objective:**

This study investigates the mediating effect of self-efficacy on the relationship between self-care ability and disability level in older adult patients with chronic diseases.

**Methods:**

A convenience sampling method was used to select 372 older adult patients with chronic diseases from five tertiary hospitals in Chengdu, Sichuan Province. General demographic information was collected using a questionnaire, and self-efficacy, self-care ability, and disability were assessed using standardized scales. Data were analyzed using SPSS 26.0, and the PROCESS macro was employed to test the mediating effect of self-efficacy.

**Results:**

The mean score for self-efficacy was 26.09 ± 7.20, for self-care ability was 113.19 ± 23.31, and for disability was 154.19 ± 29.32. Self-efficacy was positively correlated with self-care ability (*r* = 0.73, *p < 0.001.* and negatively correlated with disability (*r* = −0.84, *p < 0.001.* and self-care ability and disability (*r* = −0.91, *p < 0.001.*. The indirect effect of self-efficacy on the relationship between self-care ability and level of disability was −0.03 (95% CI −0.08 to −0.04), accounting for 16.67% of the total effect.

**Conclusion:**

Self-efficacy partially mediates the relationship between self-care ability and disability in older adult patients with chronic conditions. Healthcare providers can improve self-care behaviours and self-efficacy in older adult patients through effective interventions to reduce the incidence of disability.

## 1 Introduction

As of 2020, the population aged 60 and above constituted 18.7% of the total population in China. It is projected that by 2050, the older adult population will reach 454 million, accounting for 33% of the total population (1). The prevalence of chronic diseases among individuals aged 60 and above in China is 69.13%, with a comorbidity rate of 43.65% (2). The reduced daily activity levels and increased hospitalization rates experienced by older adult patients with chronic diseases impose significant caregiving and economic burdens on families and society. The enhancement of self-management abilities during the course of a disease has become a significant public health concern in the context of global ageing. Patients with high self-efficacy are more likely to adopt positive coping strategies when facing diseases (3), which may lead to enhanced self-care awareness and a reduced risk of disability over the course of the disease process. A number of studies have demonstrated an association between self-care (4–6), self-efficacy (7–9) and disability in certain chronic conditions. We observed that self-efficacy may act as a pivotal intermediary variable between self-care and disability among patients with chronic diseases. This notion is supported by Bandura’s Self-Efficacy Theory (10). Cheng et al. elucidated that self-efficacy and self-care are mutually reinforcing processes (10). As individual capabilities, such as self-care abilities, are enhanced, self-efficacy is also increased. Furthermore, high self-efficacy is vital for effective self-management, enabling the modification of unhealthy behaviours and outcomes, such as disability. A substantial body of research has demonstrated the interrelationship between self-efficacy, self-care ability, and chronic diseases in the older adult population. However, there is a paucity of studies that have investigated the relationship between self-efficacy, self-care ability, and disability assessment results. To the best of our knowledge, no research has hitherto investigated the relationship between these variables among older adult patients with chronic diseases in south-west China. It is imperative that this research gap be addressed, given the high prevalence of chronic diseases and disability in this population. Identifying these relationships is crucial for developing appropriate interventions aimed at reducing disability among older adult patients with chronic disease. This study aims to investigate the mediating effect of self-efficacy on the relationship between self-care ability and disability in older adult patients with chronic diseases, thereby providing a reference for rehabilitation guidance in this population.

## 2 Methods and materials

### 2.1 Study participants

Convenience sampling was employed to select patients aged 60 and above who visited the outpatient departments or were admitted to the internal medicine wards of five tertiary hospitals in Chengdu, Sichuan Province, from October 2021 to February 2022. For older individuals who utilise smartphones, a WeChat questionnaire was employed for the on-site network survey. Conversely, for older individuals who do not possess smartphones or who have no mobile phones, a paper version of the questionnaire was distributed for the on-site survey. In order to be included in the study, participants had to meet the following criteria: (1) be aged 60 and above; (2) have been diagnosed with one or more chronic diseases; (3) have a certain level of reading comprehension; and (4) provide informed consent and participate in the study voluntarily. Participants were excluded if they met any of the following criteria: (1) were critically ill; and (2) had communication difficulties due to deafness, speech impairment, or cognitive impairment.

Severe disability is defined as the presence of a large number of organ defects, significant organ malformations and moderate organ dysfunction. However, patients are considered critically ill if their vital signs are unstable, their condition is changing rapidly and more than two organ systems show signs of instability, deterioration or failure. People with severe disabilities are not the same as those in critical condition. Our study included older adult patients with chronic diseases and varying degrees of disability.

The number of severely disabled patients involved in this study is minimal. Furthermore, the severely disabled patients are non-intellectual and non-communication disabled patients, which is consistent with the inclusion and exclusion criteria of our study. In the case of severely disabled patients, the researcher will provide a detailed explanation of the purpose, content, requirements, and confidentiality of the survey, ensuring that the patient fully comprehends the nature of the investigation. Throughout the course of the study, the researcher will provide assistance to the patient should they require it.

The outpatient department is the primary location for the treatment of patients with chronic diseases. Those patients who require more intensive care are admitted to the internal medical ward. Consequently, the internal medical ward has a higher concentration of patients with chronic diseases. The objective of the study was to examine the interrelationship between self-efficacy, self-care capability and disability in older adult patients with chronic illnesses. To this end, a survey was conducted in both outpatient clinics and internal medical wards.

### 2.2 Study methods

#### 2.2.1 Research tools

##### 2.2.1.1 General demographic questionnaire

A self-designed questionnaire was employed to collate data pertaining to the patients’ gender, age, marital status, educational attainment, personal monthly income, average family income, cohabitation status, smoking and drinking habits, and the types of chronic diseases they were suffering from.

##### 2.2.1.2 General self-efficacy scale

The GSES, originally developed by Schwarzer et al. (11), was subsequently translated into Chinese by Zhang (12). The scale comprises 10 items that assess an individual’s confidence in their ability to overcome setbacks and difficulties. Each item is scored on a 4-point Likert scale, ranging from “completely incorrect” to “completely correct,” with scores ranging from 1 to 4. The sum of the scores for the 10 items represents the scale’s total score. The total score on the scale ranges from 4 to 40 points, with higher scores indicating greater self-efficacy. The Chinese version of this scale has been employed extensively across diverse contexts and has demonstrated robust psychometric properties.

##### 2.2.1.3 Exercise of self-care agency scale

The ESCA, originally developed by Kearney and Fleischer (13), was subsequently translated into Chinese by Wang et al. (14). The scale comprises four dimensions: self-care skills (items 1–12), self-care responsibility (items 13–29), self-concept (items 30–35) and health knowledge (items 36–43), with a total of 43 items. Each item is scored on a 5-point Likert scale, ranging from “completely disagree” to “completely agree,” with scores ranging from 0 to 4. Eleven items are reverse-scored. The total score ranges from 0 to 172, with higher scores indicating a greater capacity for self-care. The classification of self-care ability is determined by the total score and the scores assigned to each dimension. This results in three levels of self-care ability: low, moderate, and high. A score below 33% of the total score (57 points) indicates low-level ability, a score between 34 and 66% (58–114 points) indicates moderate-level ability, and a score above 66% of the total score (114 points) indicates high-level ability (15). The Chinese version of this scale has been employed in a multitude of contexts and has demonstrated robust psychometric properties (16, 17).

##### 2.2.1.4 Elderly disability assessment scale

The EDAS, developed by Yang et al. (18), is comprised of 28 items distributed across seven dimensions: mental function (items 1–4), organ function (items 5–7), communication (items 8–9), activity (items 10–15), self-care (items 16–23), family life (items 24–25), and economic and social life (items 26–28). Each item is assigned a score between 1 and 7, with the total score being the sum of all the scores. The total score ranges from 28 to 196, with lower scores indicating higher levels of disability. A total score below 196 indicates the presence of disability. Scores between 168 and 195 indicate mild disability, while scores between 140 and 167 indicate moderate disability. Finally, scores between 84 and 139 indicate severe disability, and a score of 28 to 83 is indicative of an extremely severe disability. The Chinese version of this scale has been employed extensively across diverse contexts and has demonstrated robust psychometric properties (19, 20). The present study is primarily concerned with a comprehensive assessment of disability and dysfunction in older adult patients with chronic diseases. Consequently, the research tool selected for this study is the EDAS.

#### 2.2.2 Data collection and quality control methods

Two survey teams were constituted, comprising four research team members from different hospital departments or units and four trained nurses. It was the responsibility of each team to collect data within their designated department or unit. The purpose, content, requirements, and confidentiality of the survey were elucidated to the patients by the investigators in accordance with the established standardized instructions. Once consent had been obtained, the questionnaires were distributed to the patients. The completed questionnaires were subsequently reviewed on-site by the investigators, collated, and the subjects were presented with a token of appreciation in the form of a small gift. Two individuals were tasked with the organisation, verification, and entry of the data, while any questionnaires deemed invalid were excluded. A total of 380 questionnaires were distributed, and 372 were returned as valid, resulting in a valid response rate of 97.89%.

#### 2.2.3 Statistical analysis

The statistical analysis was conducted using the SPSS 26.0 software. Descriptive statistics were presented as means and standard deviations for continuous variables. The independent samples *t*-test and one-way analysis of variance (ANOVA) were employed for the purpose of comparing the means between the groups in question. In the event of discrepancies in the number of items included in the dimensions under consideration, the scores are normalised and subsequently compared. Pearson’s correlation coefficient was employed to examine the relationships between variables. The PROCESS macro (Model 4) was employed to test the mediating effect of self-efficacy. Bootstrap resampling with 5,000 bootstrap samples was conducted to estimate the 95% confidence intervals for the various effects.

#### 2.2.4 Common method bias test

In order to avoid common method bias, this study employed a number of techniques to control for potential procedural influences. These included the use of anonymous surveys, reverse item settings and the presentation of items in different contexts (21). With regard to the statistical analysis, a confirmatory factor analysis was conducted using the AMOS 25.0 software. The model fit indices were as follows: The model fit indices indicated poor fit, with a χ^2^/df ratio of 4.29, an RMSEA of 0.89, a CFI of 0.79, and an NFI of 0.74. Accordingly, the potential for serious common method bias was effectively mitigated in this study.

## 3 Results

### 3.1 Demographic characteristics of the sample

Table 1 presents a summary of the demographic characteristics of the participants. The sample was predominantly outpatient, with the majority of respondents being female and over the age of 70. Of all respondents, 289 were married and 154 lived with their spouse, while 217 had completed junior high school or less. Additionally, 56% of patients had three to four chronic diseases. Significant differences in disability score between outpatients and in-patients, between males and females and across age groups, but there is no significant differences across the other study variables.

**Table 1:** Univariate analysis of scores on the disability assessment scale for older adult patients with chronic diseases (*n* = 372).

Variable	Group	Frequency	Score (Mean ± SD)	*t/F value*	*p*-value
Group	Outpatient	217	157.81 ± 23.21	14779.50^a^	0.045
	Inpatient	155	169.11 ± 35.65		
Gender	Male	163	151.21 ± 28.99	19162.50^a^	0.037
	Female	209	156.51 ± 29.43		
Age	60~	135	150.03 ± 25.92	13.715^b^	0.001
	70~	165	153.51 ± 28.11		
	80~	72	164.78 ± 35.26		
Marital status	Unmarried	4	143.75 ± 13.81	2.269^b^	0.519
	Married	289	155.06 ± 27.81		
	Divorced	7	159.71 ± 47.31		
	Widowed	72	151.56 ± 33.510		
Education level	Primary school and below	108	150.72 ± 29.59	5.101^b^	0.277
	Junior high school	109	157.74 ± 27.76		
	High school or technical school	88	152.42 ± 30.60		
	College or undergraduate	62	155.50 ± 30.21		
	Postgraduate and above	5	146.20 ± 16.97		
Monthly personal income	Below 1,000	57	157.33 ± 23.17	0.982^b^	0.417
	1,001–2000	53	151.87 ± 28.92		
	2001–3,000	99	152.71 ± 32.40		
	3,001–4,000	78	150.85 ± 29.15		
	Above 4,000	85	158.31 ± 29.63		
Monthly *per capita* household income	Below 2000	46	152.24 ± 25.01	0.742^b^	0.564
	2001–3,000	43	150.91 ± 37.41		
	3,001–4,000	69	155.01 ± 31.77		
	4,001–5,000	67	150.78 ± 29.50		
	Above 5,000	147	156.92 ± 26.61		
Living arrangement	Spouse and children	100	155.74 ± 27.50	5.220^b^	0.265
	Spouse	154	155.29 ± 26.27		
	Children	71	169.51 ± 30.87		
	Living alone	38	156.95 ± 37.47		
	Other	9	163.33 ± 45.39		
Smoking	Yes	61	150.21 ± 31.57	8787.000^a^	0.360
	No	311	154.96 ± 28.85		
Alcohol consumption	Yes	69	152.70 ± 29.52	10073.000^a^	0.635
	No	303	154.52 ± 29.32		
Type of chronic disease	1–2 diseases	160	154.52 ± 27.87	0.301^b^	0.740
	3–4 diseases	210	153.79 ± 30.53		
	5 or more diseases	2	149.50 ± 16.36		

a Univariate analysis performed using *t*-test.

b Univariate analysis performed using analysis of variance.

### 3.2 Factor analysis and internal consistency

Factor analysis was used to test the validity of the scales and the samples were randomly divided into two: one was analyzed by exploratory factor analysis (EFA) and the other was processed by confirmatory factor analysis (CFA) to confirm the factor structures of the sample obtained by EFA. *T*-test analysis was applied to both samples. The goodness of fit of the scales was assessed using the model fit indices (22), supplemented by Maione et al. (23), as shown: χ^2^ /df < 5, RMSEA <0.08, CFI > 0.90 and NFI > 0.90. In this study, Cronbach’s alpha was used to measure the reliability of the instruments.

To test the validity of the scales used in this study, the samples were randomly divided into two parts, namely General Self-Efficacy 1 (GSES 1, *N* = 186) and General Self-Efficacy 2 (GSES 2, *N* = 186). *T*-test analysis was performed, and no significant difference in group, gender, age, or other variables was observed between the two samples (all *p* > 0.05). An exploratory analysis (principal component analysis with varimax rotation) was performed to confirm the factor structure of the GSES 1 items, and a principal component was extracted explaining 68.745% of the initial variance (KMO = 0.84, Bartlett’s χ^2^ = 1,295.079, *p < 0.001.*. CFA processed GSES 2 to verify the one-factor structure of GSES 1 obtained by EFA and showed acceptable fit indices (χ^2^ /df = 3.71, RMSEA = 0.07, CFI = 0.92, NFI = 0.90). The Cronbach’s alpha for the whole scale was 0.93. Similarly, the Self-Care Agency samples were randomly divided into two parts: ESCA1 (*N* = 186) and ESCA2 (*N* = 186), *t*-test analysis indicated no statistical difference in demographic variables between these samples (all *p* > 0.05). EFA on ESCA1 revealed four principal components explaining 66.721% of the initial variance (KMO = 0.823, Bartlett’s χ^2^ = 1,724.817, *p < 0.001.*, CFA on ESCA2 showed acceptable fit indices (χ^2^/df = 2.98, RMSEA = 0.07, CFI = 0.98, NFI = 0.92). The Cronbach’s alpha for the whole scale was 0.95. The Elderly Disability Assessment samples were also randomly split in two: EDAS1 (*N* = 186) and EDAS2 (*N* = 186), *t*-test analysis indicated no significant difference in demographic variables between the two samples (all *p* > 0.05). EFA on EDAS1 suggested a seven dimensional structure explaining 71.657% of the initial variance (KMO = 0.771, Bartlett’s χ^2^ = 181.469, *p < 0.001.*. The CFA on EDAS2 showed good goodness of fit (χ^2^/df = 3.68, RMSEA = 0.08, CFI = 0.97, NFI = 0.91). The Cronbach’s alpha was 0.90.

### 3.3 Robustness analysis

In order to test the robustness of this study, we divided the sample into a male group (*n* = 163) and a female group (*n* = 209). The results are shown in Tables 2, 3. The results suggest that the total effect of self-care ability on disability level was −0.18 (95% CI -0.20 to −0.16) for the male group and − 0.19 (95% CI -0.25 to −0.16) for the female group. Minor changes are observed between the two groups, but the interpretation of the results remains essentially unchanged.

**Table 2:** Mediating effect of self-efficacy on the relationship between self-care ability and disability level for the male group (*n* = 163).

Effect type	Effect value	*SE*	95%*CI*	Mediating effect
Total effect	−0.18	0.03	−0.20 ~ −0.16	
Direct effect	−0.15	0.03	−0.16 ~ −0.09	83.33%
Indirect effect	−0.03	0.01	−0.08 ~ −0.01	16.67%

**Table 3:** Mediating effect of self-efficacy on the relationship between self-care ability and disability level for the female group (*n* = 209).

Effect type	Effect value	*SE*	95%*CI*	Mediating effect
Total effect	−0.19	0.03	−0.25 ~ −0.16	
Direct effect	−0.17	0.02	−0.23 ~ −0.08	89.47%
Indirect effect	−0.02	0.01	−0.09 ~ −0.05	10.53%

### 3.4 Scale scores

Table 4 presents the scores for self-efficacy, self-care ability and disability. The self-efficacy score of older adult patients with chronic diseases was found to be (26.09 ± 7.20), with a range of 10 to 40. The score for self-care ability was (113.19 ± 23.31), with a range of 44 to 176. The disability level score was (154.19 ± 29.32), with a range of 52 to 260. These results indicate that all three variables are within the moderate level. In terms of self-care ability, 73 individuals (19.62%) demonstrated a low level of ability, while 215 individuals (57.80%) exhibited a medium level of ability. Conversely, 84 individuals (22.58%) exhibited a high level of ability. Furthermore, with regard to disability, a total of 141 individuals (37.90%) demonstrated a mild disability level, while 226 individuals (60.75%) exhibited a moderate disability level. In contrast, five individuals (1.34%) exhibited a severe disability level. The dimensions of self-care ability were found to vary in terms of their respective scores. The dimension of self-care skills (0.76) and the dimension of health knowledge (0.91) exhibited the highest Z-scores. The observed result may be attributed to the fact that the subjects of this study originate from Chengdu, a city with a relatively developed economy and an extensive social support system. These factors may have facilitated the acquisition of self-care skills and health knowledge among older adult patients with chronic diseases. In this study, the communication dimension (0.15) and the family life dimension (0.11) exhibited the lowest Z-scores among the seven dimensions of disability. This may be attributed to the influence of traditional Chinese culture and the stigma associated with it. Older adult patients with chronic diseases are often reluctant to engage in active communication with the outside world. Furthermore, contemporary Chinese adult children experience significant pressure in their professional and personal lives. They tend to prioritize the needs of the next generation over those of the previous generation, which can lead to a lack of interaction and communication with their parents. This lack of communication can have a detrimental impact on the internal stability of family relationships.

**Table 4:** Self-efficacy, self-care ability, and disability assessment scores (*n* = 372).

Scale and dimension	Score (Mean ± SD)	Z-scores
**Self-efficacy**	26.09 ± 7.20	
**Total self-care ability**	113.19 ± 23.31	
Self-care skill	34.95 ± 7.65	0.76
Self-care responsibility	12.41 ± 3.58	0.34
Self-care concept	17.56 ± 5.02	0.65
Health knowledge level	48.28 ± 9.32	0.91
**Total older adult disability assessment score**	154.19 ± 29.32	
Mental function	19.24 ± 4.46	0.26
Organ function	14.02 ± 4.93	0.19
Communication	12.44 ± 2.07	0.15
Activity	32.22 ± 7.53	0.79
Self-care	45.92 ± 8.53	0.87
Family life	12.01 ± 2.98	0.11
Economic and social life	18.34 ± 4.05	0.22

### 3.5 Bivariate analysis

As illustrated in Table 5, a positive correlation was identified between self-efficacy and self-care ability (*r* = 0.73, *p < 0.001.*, whereas a negative correlation was observed between self-efficacy and level of disability (*r* = −0.84, *p < 0.001.*. Similarly, a negative correlation was identified between self-care ability and level of disability (*r* = −0.91, *p < 0.001.*. The findings suggest that elevated levels of self-care ability and self-efficacy in older patients are associated with a reduction in disability.

**Table 5:** Correlation analysis of self-efficacy, self-care ability, and disability assessment (*n* = 372).

Item	Self-efficacy	Self-care ability	Self-care skills	Self-care responsibility	Self-care concept	Health knowledge	Older adult disability	Assessment mental function	Organ function	Communication	Activity	Self-care	Family life	Economic and social
Self-efficacy	1.00	0.73^a^	0.60^a^	0.62^a^	0.66^a^	0.69^a^	-0.84^a^	0.004	−0.07	0.06	−0.04	−0.06	0.08	0.08
Self-care Ability	0.73^a^	1.00	0.93^a^	0.89^a^	0.87^a^	0.95^a^	−0.91^a^	0.06	0.03	0.12	−0.002	0.02	0.08	0.08
Self-care skills	0.60^a^	0.93^a^	1.00	0.80^a^	0.75^a^	0.83^a^	0.07	0.07	0.06	0.13	0.03	0.04	0.10	0.09
Self-care Responsibility	0.62^a^	0.89^a^	0.80^a^	1.00	0.75^a^	0.83^a^	0.02	0.05	0.02	0.09	−0.03	0.03	0.05	0.05
Self-care concept	0.66^a^	0.87^a^	0.75^a^	0.75^a^	1.00	0.78^a^	0.03	0.02	−0.02	0.09	−0.02	0.06	0.09	0.07
Health knowledge	0.69^a^	0.95^a^	0.83^a^	0.83^a^	0.78^a^	1.00	0.03	0.06	0.02	0.09	−0.03	−0.02	0.06	0.07
Older adult disability	−0.84^a^	−0.91^a^	0.07	0.02	0.03	0.03	1.00	0.81^a^	0.80^a^	0.86^a^	0.89^a^	0.85^a^	0.84^a^	0.85^a^
Assessment mental function	0.004	0.06	0.07	0.05	0.02	0.06	0.81^a^	1.00	0.71^a^	0.69^a^	0.61^a^	0.56^a^	0.54^a^	0.56^a^
Organ function	−0.07	0.03	0.06	0.02	−0.018	0.02	0.80^a^	0.71^a^	1.00	0.73^a^	0.72^a^	0.67^a^	0.69^a^	0.61^a^
Communication	0.06	0.12	0.13	0.01	0.09	0.09	0.86^a^	0.69^a^	0.73^a^	1.00	0.78^a^	0.75^a^	0.75^a^	0.73^a^
Activity	−0.04	−0.002	0.03	−0.03	−0.02	−0.03	0.89^a^	0.61^a^	0.72^a^	0.78^a^	1.00	0.85^a^	0.81^a^	0.81^a^
Self-care	−0.06	0.02	0.04	0.03	0.03	−0.02	0.85^a^	0.56^a^	0.67^a^	0.75^a^	0.85^a^	1.00	0.83^a^	0.82^a^
Family life	0.08	0.08	0.10	0.05	0.09	0.06	0.84^a^	0.54^a^	0.61^a^	0.75^a^	0.81^a^	0.83^a^	1.00	0.89^a^
Economic and social	0.08	0.08	0.09	0.05	0.07	0.07	0.85^a^	0.56^a^	0.61^a^	0.73^a^	0.81^a^	0.82^a^	0.89^a^	1.00

a *p < 0.001.*

### 3.6 Mediation effect analysis

The PROCESS programme was employed to test the mediating effect of self-efficacy on the relationship between self-care ability and disability level. In this analysis, self-care ability was designated as the independent variable, self-efficacy as the mediating variable, and disability level as the dependent variable, and all demographic variables in Table 1 as the control variables. As illustrated in Table 6, self-care ability was found to have a significant negative predictive effect on disability level (β = −0.19, *p < 0.001.*, while self-care ability also demonstrated a positive predictive effect on self-efficacy (β = 0.24, *p < 0.001.*. Upon entering both self-care ability and self-efficacy into the regression equation simultaneously, both self-care ability (β = −0.16, *p < 0.001.* and self-efficacy (β = −0.17, *p < 0.001.* remained significant predictors of disability level. Table 7 illustrates that the overall effect of self-care ability on disability level was −0.18 (95% CI −0.29 to −0.14). The indirect effect of self-efficacy on the relationship between self-care ability and level of disability was −0.03 (95% CI −0.08 to −0.04), which did not include zero, indicating that self-efficacy partially mediated the relationship between self-care ability and level of disability.

**Table 6:** Regression analysis of the relationships between self-care ability, self-efficacy, and disability level variables (*n* = 372).

Regression equation		Fit indices				
Outcome variable	Predictor	*R*	*R* ^2^	*F*	*β*	*t*
Disability level	Self-care ability	0.18	0.03	27.71^a^	−0.19	−5.20^a^
Self-efficacy	Self-care ability	0.24	0.05	43.19^a^	0.24	6.59^a^
Disability level	Self-care ability	0.25	0.06	25.19^a^	−0.16	−3.38^a^
	Self-efficacy				−0.17	−4.55^a^

a *p < 0.001.*

**Table 7:** Mediating effect of self-efficacy on the relationship between self-care ability and disability level (*n* = 372).

Effect type	Effect value	*SE*	95%*CI*	Mediating effect
Total effect	−0.18	0.03	−0.29 ~ −0.14	
Direct effect	−0.15	0.03	−0.18 ~ −0.06	83.33%
Indirect effect	−0.03	0.01	−0.08 ~ −0.04	16.67%

## 4 Discussion

This study represents the first attempt to examine the relationship between self-care, self-efficacy and disability among older adult patients with chronic diseases in Southwest China. It also examines whether self-efficacy mediates the relationship between self-care and disability in this population. Several significant findings emerged from this study (Table 8).

**Table 8:** Univariate analysis of scores on the self-care assessment scale for older adult patients with chronic diseases (*n* = 372).

Variable	Group	Frequency	Score (Mean ± SD)	*t/F value*	*p*-value
Group	Outpatient	217	117.03 ± 15.67	8856.791^a^	0.041
	Inpatient	155	123.34 ± 18.54		
Gender	Male	163	113.89 ± 19.26	9012.332^a^	0.039
	Female	209	121.54 ± 20.68		
Age	60~	135	130.47 ± 18.65	8.943^b^	0.008
	70~	165	126.41 ± 17.83		
	80~	72	109.95 ± 13.52		
Marital status	Unmarried	4	124.55 ± 17.49	4.071^b^	0.217
	Married	289	127.01 ± 18.59		
	Divorced	7	106.48 ± 21.04		
	Widowed	72	116.83 ± 16.98		
Education level	Primary school and below	108	120.59 ± 17.33	6.540^b^	0.013
	Junior high school	109	108.46 ± 21.01		
	High school or technical school	88	105.69 ± 15.78		
	College or undergraduate	62	112.36 ± 20.15		
	Postgraduate and above	5	116.20 ± 17.38		
Monthly personal income	Below 1,000	57	117.53 ± 19.08	1.237^b^	0.572
	1,001–2000	53	121.44 ± 21.04		
	2001–3,000	99	108.76 ± 15.89		
	3,001–4,000	78	121.50 ± 17.41		
	Above 4,000	85	118.49 ± 17.59		
Monthly *per capita* household income	Below 2000	46	125.77 ± 20.12	1.526^b^	0.487
	2001–3,000	43	106.49 ± 16.20		
	3,001–4,000	69	102.95 ± 19.65		
	4,001–5,000	67	127.14 ± 22.38		
	Above 5,000	147	121.89 ± 17.88		
Living arrangement	Spouse and children	100	104.45 ± 18.90	3.479^b^	0.284
	Spouse	154	115.25 ± 22.19		
	Children	71	106.43 ± 17.52		
	Living alone	38	114.87 ± 20.53		
	Other	9	113.35 ± 17.41		
Smoking	Yes	61	125.05 ± 18.02	7539.237^a^	0.050
	No	311	104.67 ± 15.80		
Alcohol consumption	Yes	69	112.43 ± 16.26	8347.118^a^	0.048
	No	303	102.71 ± 20.30		
Type of chronic disease	1–2 diseases	160	121.59 ± 18.41	0.481^b^	0.683
	3–4 diseases	210	107.61 ± 17.85		
	5 or more diseases	2	117.88 ± 15.49		

a Univariate analysis performed using *t*-test.

b Univariate analysis performed using analysis of variance.

### 4.1 Self-care ability, general self-efficacy, and disability level in older adult patients with chronic diseases

Self-care capability refers to engaging in self-care activities or self-management using various resources (24). It can be acquired through learning, and forms the basis for individuals to engage in self-care behaviours that promote and maintain their physical and mental health and improve their quality of life (25). Self-care capability is an important indicator for assessing patients’ self-care behaviours, and its level can influence their health and quality of life. Research has shown that patients with higher self-care ability have greater confidence in managing their condition, better quality of life after discharge from hospital, and better disease outcomes (26). The results of this study showed that the self-care ability score of older adult patients with chronic diseases was moderate (113.19 ± 23.31), which is consistent with similar studies in China (27) and higher than that of inpatients/cancer/chronic obstructive pulmonary disease (COPD) patients (28, 29). As previously stated, the dimensions of self-care skills and health knowledge exhibited the highest scores. The theory of successful ageing emphasises that through effective self-health management, older adults can accept themselves, actively learn, participate in the life process and achieve a sense of accomplishment. As we mentioned above, the study population in this research was from Chengdu, a region in the central part of Sichuan Province with a well-established service network, comfortable living environment, and abundant cultural and sports activities. Under the advocacy of the local government, various levels of society are actively promoting the construction of an “active ageing” cultural environment that integrates “cultural and creative” elements, and are actively carrying out support services and health education activities for older adult patients with chronic diseases. As a result, older adult patients with chronic diseases can accept themselves and actively learn as they age, which is conducive to maintaining their self-care ability and self-health management (Table 9).

**Table 9:** Univariate analysis of scores on the self-efficacy assessment scale for older adult patients with chronic diseases (*n* = 372).

Variable	Group	Frequency	Score (Mean ± SD)	*t/F value*	*p*-value
Group	Outpatient	217	23.57 ± 8.66	236.41^a^	0.022
	Inpatient	155	18.43 ± 10.52		
Gender	Male	163	20.55 ± 9.43	173.88^a^	0.041
	Female	209	16.76 ± 11.36		
Age	60~	135	19.56 ± 13.21	4.892^b^	0.008
	70~	165	17.43 ± 9.44		
	80~	72	15.42 ± 13.88		
Marital status	Unmarried	4	23.47 ± 12.89	3.541^b^	0.012
	Married	289	25.07 ± 13.60		
	Divorced	7	19.45 ± 7.99		
	Widowed	72	21.43 ± 9.41		
Education level	Primary school and below	108	20.76 ± 11.02	6.902^b^	0.006
	Junior high school	109	25.48 ± 9.02		
	High school or technical school	88	21.49 ± 8.64		
	College or undergraduate	62	20.14 ± 10.52		
	Postgraduate and above	5	18.78 ± 6.98		
Monthly personal income	Below 1,000	57	20.38 ± 9.42	1.429^b^	0.433
	1,001–2000	53	29.13 ± 11.42		
	2001–3,000	99	30.58 ± 12.37		
	3,001–4,000	78	28.76 ± 14.48		
	Above 4,000	85	23.79 ± 13.70		
Monthly *per capita* household income	Below 2000	46	25.45 ± 15.11	0.679^b^	0.567
	2001–3,000	43	27.34 ± 9.89		
	3,001–4,000	69	25.67 ± 11.08		
	4,001–5,000	67	23.41 ± 9.63		
	Above 5,000	147	26.54 ± 10.58		
Living arrangement	Spouse and children	100	21.59 ± 9.59	4.671^b^	0.009
	Spouse	154	17.83 ± 6.78		
	Children	71	19.62 ± 10.49		
	Living alone	38	16.35 ± 7.40		
	Other	9	20.48 ± 9.33		
Smoking	Yes	61	19.59 ± 11.32	232.116^a^	0.020
	No	311	17.39 ± 9.04		
Alcohol consumption	Yes	69	21.40 ± 9.53	378.450^a^	0.009
	No	303	28.37 ± 11.39		
Type of chronic disease	1–2 diseases	160	24.11 ± 7.99	0.471^b^	0.610
	3–4 diseases	210	23.84 ± 10.14		
	5 or more diseases	2	19.66 ± 8.51		

a Univariate analysis performed using *t*-test.

b Univariate analysis performed using analysis of variance.

Self-efficacy is an individual’s assessment of their ability to perform certain behaviours. A strong perceptual and motivational source drives individuals to overcome challenges and ultimately achieve success (30). It represents an individual’s overall confidence in coping with various environmental challenges or new situations, and serves as an important resource for dealing with the external world. Improving self-efficacy can improve medication adherence, enhance self-management and self-care skills, reduce hospital readmission rates, lower healthcare costs, modulate psychological states, improve adverse outcomes, and ultimately improve the quality of life of patients with chronic diseases (31–33). Studies have shown that self-efficacy predicts medication adherence in older adult hypertensive patients and moderates the relationship between depression and medication adherence (34). In a study of 153 asymptomatic patients with heart failure, gratitude was correlated with self-efficacy, and self-efficacy was positively correlated with medication adherence. Gratitude influences medication adherence indirectly through self-efficacy (31). The results of this study showed that the self-efficacy score of older adult patients with chronic diseases was moderate (26.09 ± 7.20), which is similar to several other studies (35–37), indicating a relatively low proactive and positive attitude of older adult patients with chronic diseases in adapting to society and managing their health. The reasons for this may be that older people with chronic diseases have difficulties adjusting to their roles after diagnosis and have low acceptance of their disease, which affects their subjective assessment of their own abilities. During the illness, their daily activity level decreases, which increases the likelihood and frequency of hospitalization, which places a burden on their families and society, as well as an economic burden. This can lead to a decline in their self-esteem and self-confidence, resulting in self-doubt and lower self-efficacy. Four types of information influence the formation and change of self-efficacy: personal experiences of success or failure, vicarious reinforcement through observation, evaluation and persuasion by others, and emotional and physiological information (38). Healthcare providers can encourage older people with chronic diseases to develop regular exercise habits, implement effective health education, establish a multidisciplinary self-management approach led by nurses, and use telemedicine (39) to improve their self-efficacy and self-management skills, thereby enhancing their quality of life and promoting their health.

Disability is a condition in which an individual’s ability to function in daily life or social activities is limited (40). Prolonged disability can cause severe psychological trauma to patients, leading to psychological fragility, role dysfunction, adverse treatment outcomes, and impairing the recovery process (41). Disability in the older adult population is directly related to care practices and costs, resulting in a significant long-term burden of care (42). The results of this survey showed that the disability score of older adult patients with chronic diseases was moderate (174.19 ± 29.32), which is consistent with the research results of Wu (43), Zhang (44), and others. The lowest scores were observed in the communication and family life dimensions in this study. Possible factors contributing to this analysis include: cognitive decline, physiological ageing, shrinking social networks, and rapid changes in economic conditions and social roles, among others. Taken together, these elements challenge older patients with chronic conditions to learn and accept new concepts. Their thoughts and behaviours become relatively closed, influenced by traditional Chinese culture and the stigma associated with illness. They tend to avoid contact and communication with others for fear of discrimination. This lack of social interaction and support impairs their ability to adapt and receive support from family members, friends and social groups, which is detrimental to intimacy and reciprocity within the family. Chronically ill older people who cannot care for themselves often choose to live with their adult children (45). They experience feelings of loneliness, which leads to long-term and severe social and psychological stress, and reduces their access to social and emotional support (46). However, with rapid social and economic development, the migration of adult children has a negative impact on older people, resulting in a lack of support and care from family, friends and society, which is not conducive to the stability of intimate family relationships and mutual support. This can lead to feelings of isolation and severe social and psychological pressures. Therefore, it is recommended to improve the social support system with a focus on disabled older people with chronic diseases, to enhance social integration and to encourage adult children to understand the needs of their parents in order to effectively guarantee the quality of life of older people and to create a harmonious and healthy family atmosphere, thus promoting the stable functioning of family dynamics (47). Standardising long-term care programmes for disabled older people (48) and exploring service models suitable for long-term care of older people with chronic diseases can effectively improve their self-efficacy and self-management skills, thereby improving their physical and mental health and quality of life.

### 4.2 Correlation analysis of general self-efficacy, self-care ability, and disability level in older adult patients with chronic diseases

The results of this study showed a positive correlation between self-efficacy and self-care ability (*r* = 0.73, *p < 0.001.*, which is consistent with previous studies (49, 50). Patients with higher self-efficacy may have greater self-confidence, are active learners, improve their knowledge and skills to manage their disease, and improve their self-care skills. It is also conceivable that individuals who demonstrate superior self-care abilities are more prone to exhibit elevated levels of self-efficacy. Conversely, patients with low self-efficacy have less confidence, are unable to improve their self-care skills through psychological counselling, and have poor self-management behaviours when faced with illness. In addition, a negative correlation was found between self-efficacy and level of disability (*r* = −0.84, *p < 0.001.*, which is consistent with research by Marceron et al. (51). Older adults with higher self-efficacy have a better understanding of their health conditions, have better emotional management skills (52), have confidence and skills to cope with illness, are more adaptable to changes in social and family roles after illness, actively seek medical and family support, and have positive expectations for their future lives. As a result, they effectively regulate anxiety, depression and other negative emotions. This suggests that health care providers should increase psychological interventions for patients, improve patients’ self-efficacy in managing their condition, and consequently reduce levels of disability. In addition, there was a negative correlation between self-care ability and level of disability (*r* = −0.91, *p < 0.001.*, indicating that patients with higher self-care ability have lower levels of disability. Self-care theory suggests that self-care ability can meet self-care needs through scientific and rational guidance and learning, thereby improving quality of life and prognosis (53). This means that older adult patients with higher self-care capacity can use internal and external resources, actively participate in decision making, fully utilise their subjective initiative, realise their self-worth, increase their confidence in self-care, improve their self-care ability, and maintain their physical health, prevent disease and facilitate disease recovery, thus reducing the level of disability.

### 4.3 Partial mediating role of self-efficacy in the relationship between self-care ability and disability level in older adult patients with chronic diseases

The mediation analysis showed that self-efficacy partially mediated the relationship between self-care ability and level of disability. Self-care theory (54) suggests that older adults can cultivate their self-care ability and environmental adaptability through late learning of self-care behaviours, which allows them to demonstrate positive emotions such as self-esteem, wisdom, self-efficacy and self-control when faced with symptoms (55). Older adults with higher self-care skills typically integrate and utilise resources around them to effectively cope with adverse life events such as various chronic diseases, widowhood and living alone (56), thereby improving their quality of life. The results of this study further support self-care theories and Bandura’s self-efficacy theory as discussed above. Older patients with higher self-care abilities are more confident and inclined to proactively use internal and external resources, acquire relevant knowledge and skills, and apply them to manage their own physical and emotional well-being. They actively cope with adverse life events, experience growth and adaptation as they age, achieve new equilibria, and subsequently reduce levels of disability.

On the other hand, older patients with lower self-care capacity tend to use avoidance strategies when dealing with illness, show poor self-management behaviours, lack practical coping skills when faced with illness, and experience greater uncertainty about their health status, resulting in higher levels of disability. Previous studies have also shown that self-care skills are beneficial for self-behavioural management and emotional regulation in chronic illness (57). Older adults with inadequate self-care skills have significantly higher rates of disability and disease burden, placing a significant burden on families, society and health care institutions. Self-care ability is a modifiable factor in self-management behaviour and can be improved through nursing interventions and various training methods. Therefore, healthcare providers can enhance the self-care capabilities of older people with chronic diseases by providing individualised interventions, improving their perceived self-efficacy and increasing their confidence in self-management. This can ultimately improve their quality of life and delay or even reverse adverse health outcomes.

### 4.4 Implications for practice

Our study provides two ideas for future research and public health practice. First, improving the self-care capabilities and self-efficacy of older people with chronic conditions is an important way to achieve self-management of chronic conditions in this population. Studies have shown that stimulating the potential of older people can effectively reduce the incidence of disability (58). Active self-management can motivate older people to actively cope with the role of ageing and to acquire knowledge and skills for self-health management. Ravesloot et al. (59) developed a management plan for people with disabilities that focuses on helping participants to set up healthy self-management courses and to build participants’ confidence in changing healthy behaviours. Since its inception in 1995, the programme has provided services to approximately 8,900 individuals at 279 community agencies across 46 states in the United States. The management programme served people through multiple levels of intervention, meeting the individual needs of participants and improving their functional status. Therefore, in China’s current “geriatric-centred” policy implementation environment, enabling older adult patients with chronic diseases to change from passive disease self-management to active participation in disease self-management can improve their self-care ability and self-efficacy to cope with disease management and actively delay disability. Second, the availability of medical resources and timely access to practical medical help are crucial for older people with chronic conditions. The incidence of disability is closely related to health services. A study of the relationship between health-related services and disability showed that the more medical services the participants used, the less likely they were to be disabled. In particular, participants who participated in leisure activities, had regular health checks and received information support were less likely to be disabled, and their disability rate was reduced by 59 to 89% (60). Effective medical services can reduce the incidence of disability, particularly in older people with chronic conditions, but current utilisation of the medical service system is not optimistic. Moreover, in some areas of southwest China, the availability of medical resources and the affordability of medical expenses have become the limiting factors for older adult patients with chronic diseases to seek medical care. Yen et al. (61) studied the use of preventive health care services among people with intellectual disability, and the results showed that only 16.65% of people with intellectual disability over 40 years old used preventive health care services, of which 19.38% used preventive health care services for mild disability and 13.83% for severe disability. As the degree of intellectual disability increases, the use of preventive health services decreases. Therefore, future studies need to combine government policy interventions and personal participation in the medical service system to effectively improve the quality of life of older people with chronic diseases and reduce the incidence of disability.

### 4.5 Limitations

This study has three major limitations. First, it is a cross-sectional study, which inherently limits the ability to establish causality, despite the theoretical framework. Meanwhile, it is not possible to exclude the possibility of an inverse causal relationship. Future studies should consider using experimental or longitudinal designs to elucidate causal relationships between variables. Second, data collection was limited to five tertiary hospitals in Chengdu, Sichuan Province, which may limit the generalisability of the findings. Third, in future work, we should further consider the influencing factors of older patients with chronic diseases, such as psychological resilience, perceived social support, disease burden and other variables, to enrich the research model.

## 5 Conclusion

Self-efficacy partially mediates the relationship between self-care ability and disability in older people with chronic diseases. Self-care ability may directly predict level of disability and indirectly predict disability through self-efficacy. Healthcare providers can implement personalised care strategies and interventions that involve older adults in their disease management, strengthen health coaching, encourage self-care behaviours and promote self-efficacy. These efforts can ultimately improve quality of life and delay the onset of disability.

## Data Availability

The raw data supporting the conclusions of this article will be made available by the authors, without undue reservation.
